# Supplementary material for: Epidemiology of congenital heart defects in France from 2013 to 2022 using the PMSI-MCO (French Medical Information System Program in Medicine, Surgery, and Obstetrics) database
Source: PLoS One. 2024 Apr 16;19(4):e0298234. doi: 10.1371/journal.pone.0298234 (PMC11020754; doi:10.1371/journal.pone.0298234)
Supplement: S1 Table — (DOCX) [file pone.0298234.s001.docx]

**S1 Table. Incidence of congenital heart defect in non-metropolitan France from 2013 to 2022 in the PMSI-MCO (French Medical Information System Program in Medicine, Surgery and Obstetrics) database according to the ICD-10 (International Statistical Classification of Diseases – 10^th^ Revision).**

| **ICD-10** |  | **Unique diagnosis** | | **Multiple diagnoses** | | **Total** | |
| --- | --- | --- | --- | --- | --- | --- | --- |
|  | **Number of patients** | **3,550** | **72.75%** | **1,330** | **27.25%** | **4,880** | **100.00%** |
| **Q20** | **Congenital malformations of cardiac chambers and connections** | **55** | **1.55%** | **386** | **29.02%** | **441** | **9.04%** |
| Q200 | Common arterial trunk | 5 | 0.14% | 28 | 2.11% | 33 | 0.68% |
| Q201 | Double outlet right ventricle | 5 | 0.14% | 73 | 5.49% | 78 | 1.60% |
| Q202 | Double outlet left ventricle | 0 | 0.00% | 11 | 0.83% | 11 | 0.23% |
| Q203 | Discordant ventriculoarterial connection | 15 | 0.42% | 93 | 6.99% | 108 | 2.21% |
| Q204 | Double inlet ventricle | 5 | 0.14% | 60 | 4.51% | 65 | 1.33% |
| Q205 | Discordant atrioventricular connection | 0 | 0.00% | 22 | 1.65% | 22 | 0.45% |
| Q206 | Isomerism of atrial appendages | 0 | 0.00% | 10 | 0.75% | 10 | 0.20% |
| Q208 | Other congenital malformations of cardiac chambers and connections | 20 | 0.56% | 62 | 4.66% | 82 | 1.68% |
| Q209 | Congenital malformation of cardiac chambers and connections. unspecified | 5 | 0.14% | 27 | 2.03% | 32 | 0.66% |
| **Q21** | **Congenital malformations of cardiac septa** | **1,149** | **32.37%** | **1,540** | **115.79%** | **2,689** | **55.10%** |
| Q210 | Ventricular septal defect | 421 | 11.86% | 602 | 45.26% | 1,023 | 20.96% |
| Q211 | Atrial septal defect | 609 | 17.15% | 612 | 46.02% | 1,221 | 25.02% |
| Q212 | Atrioventricular septal defect | 41 | 1.15% | 155 | 11.65% | 196 | 4.02% |
| Q213 | Tetralogy of Fallot | 60 | 1.69% | 125 | 9.40% | 185 | 3.79% |
| Q214 | Aortopulmonary septal defect | 4 | 0.11% | 13 | 0.98% | 17 | 0.35% |
| Q218 | Other congenital malformations of cardiac septa | 11 | 0.31% | 26 | 1.95% | 37 | 0.76% |
| Q219 | Congenital malformation of cardiac septum. unspecified | 3 | 0.08% | 7 | 0.53% | 10 | 0.20% |
| **Q22** | **Congenital malformations of pulmonary and tricuspid valves** | **52** | **1.46%** | **327** | **24.59%** | **379** | **7.77%** |
| Q220 | Pulmonary valve atresia | 1 | 0.03% | 75 | 5.64% | 76 | 1.56% |
| Q221 | Congenital pulmonary valve stenosis | 32 | 0.90% | 101 | 7.59% | 133 | 2.73% |
| Q222 | Congenital pulmonary valve insufficiency | 3 | 0.08% | 13 | 0.98% | 16 | 0.33% |
| Q223 | Other congenital malformations of pulmonary valve | 2 | 0.06% | 26 | 1.95% | 28 | 0.57% |
| Q224 | Congenital tricuspid stenosis | 1 | 0.03% | 29 | 2.18% | 30 | 0.61% |
| Q225 | Ebstein anomaly | 8 | 0.23% | 8 | 0.60% | 16 | 0.33% |
| Q226 | Hypoplastic right heart syndrome | 1 | 0.03% | 42 | 3.16% | 43 | 0.88% |
| Q228 | Other congenital malformations of tricuspid valve | 3 | 0.08% | 27 | 2.03% | 30 | 0.61% |
| Q229 | Congenital malformation of tricuspid valve. unspecified | 1 | 0.03% | 6 | 0.45% | 7 | 0.14% |
| **Q23** | **Congenital malformations of aortic and mitral valves** | **55** | **1.55%** | **184** | **13.83%** | **239** | **4.90%** |
| Q230 | Congenital stenosis of aortic valve | 0 | 0.00% | 21 | 1.58% | 21 | 0.43% |
| Q231 | Congenital insufficiency of aortic valve | 11 | 0.31% | 29 | 2.18% | 40 | 0.82% |
| Q232 | Congenital mitral stenosis | 0 | 0.00% | 18 | 1.35% | 18 | 0.37% |
| Q233 | Congenital mitral insufficiency | 24 | 0.68% | 52 | 3.91% | 76 | 1.56% |
| Q234 | Hypoplastic left heart syndrome | 15 | 0.42% | 39 | 2.93% | 54 | 1.11% |
| Q238 | Other congenital malformations of aortic and mitral valves | 5 | 0.14% | 18 | 1.35% | 23 | 0.47% |
| Q239 | Congenital malformation of aortic and mitral valves. unspecified | 0 | 0.00% | 7 | 0.53% | 7 | 0.14% |
| **Q24** | **Other congenital malformations of heart** | **301** | **8.48%** | **510** | **38.35%** | **811** | **16.62%** |
| Q240 | Dextrocardia | 13 | 0.37% | 17 | 1.28% | 30 | 0.61% |
| Q241 | Laevocardia | 2 | 0.06% | 20 | 1.50% | 22 | 0.45% |
| Q242 | Cor triatriatum | 1 | 0.03% | 8 | 0.60% | 9 | 0.18% |
| Q243 | Pulmonary infundibular stenosis | 1 | 0.03% | 27 | 2.03% | 28 | 0.57% |
| Q244 | Congenital subaortic stenosis | 1 | 0.03% | 20 | 1.50% | 21 | 0.43% |
| Q245 | Malformation of coronary vessels | 17 | 0.48% | 41 | 3.08% | 58 | 1.19% |
| Q246 | Congenital heart block | 4 | 0.11% | 5 | 0.38% | 9 | 0.18% |
| Q248 | Other specified congenital malformations of heart | 150 | 4.23% | 183 | 13.76% | 333 | 6.82% |
| Q249 | Congenital malformation of heart. unspecified | 112 | 3.15% | 189 | 14.21% | 301 | 6.17% |
| **Q25** | **Congenital malformations of great arteries** | **1,921** | **54.11%** | **1,081** | **81.28%** | **3,002** | **61.52%** |
| Q250 | Patent ductus arteriosus | 1,818 | 51.21% | 522 | 39.25% | 2,340 | 47.95% |
| Q251 | Coarctation of aorta | 25 | 0.70% | 120 | 9.02% | 145 | 2.97% |
| Q252 | Atresia of aorta | 0 | 0.00% | 12 | 0.90% | 12 | 0.25% |
| Q253 | Stenosis of aorta | 1 | 0.03% | 24 | 1.80% | 25 | 0.51% |
| Q254 | Other congenital malformations of aorta | 19 | 0.54% | 64 | 4.81% | 83 | 1.70% |
| Q255 | Atresia of pulmonary artery | 2 | 0.06% | 70 | 5.26% | 72 | 1.48% |
| Q256 | Stenosis of pulmonary artery | 29 | 0.82% | 119 | 8.95% | 148 | 3.03% |
| Q257 | Other congenital malformations of pulmonary artery | 14 | 0.39% | 64 | 4.81% | 78 | 1.60% |
| Q258 | Other congenital malformations of great arteries | 9 | 0.25% | 54 | 4.06% | 63 | 1.29% |
| Q259 | Congenital malformation of great arteries. unspecified | 4 | 0.11% | 32 | 2.41% | 36 | 0.74% |
| **Q26** | **Congenital malformations of great veins** | **17** | **0.48%** | **122** | **9.17%** | **139** | **2.85%** |
| Q260 | Congenital stenosis of vena cava | 0 | 0.00% | 1 | 0.08% | 1 | 0.02% |
| Q261 | Persistent left superior vena cava | 2 | 0.06% | 13 | 0.98% | 15 | 0.31% |
| Q262 | Total anomalous pulmonary venous connection | 2 | 0.06% | 30 | 2.26% | 32 | 0.66% |
| Q263 | Partial anomalous pulmonary venous connection | 2 | 0.06% | 11 | 0.83% | 13 | 0.27% |
| Q264 | Anomalous pulmonary venous connection. unspecified | 1 | 0.03% | 29 | 2.18% | 30 | 0.61% |
| Q265 | Anomalous portal venous connection | 0 | 0.00% | 1 | 0.08% | 1 | 0.02% |
| Q266 | Portal vein-hepatic artery fistula | 5 | 0.14% | 4 | 0.30% | 9 | 0.18% |
| Q268 | Other congenital malformations of great veins | 5 | 0.14% | 29 | 2.18% | 34 | 0.70% |
| Q269 | Congenital malformation of great vein. unspecified | 0 | 0.00% | 4 | 0.30% | 4 | 0.08% |
