# Supplementary material for: Epidemiology of congenital heart defects in France from 2013 to 2022 using the PMSI-MCO (French Medical Information System Program in Medicine, Surgery, and Obstetrics) database
Source: PLoS One. 2024 Apr 16;19(4):e0298234. doi: 10.1371/journal.pone.0298234 (PMC11020754; doi:10.1371/journal.pone.0298234)
Supplement: S2 Table — (DOCX) [file pone.0298234.s002.docx]

**S2 Table. Frequency of perinatal data between children with congenital heart defects and children without congenital malformation in non-metropolitan France from 2013 to 2022.**

| **Studied factor** | | | **Patients with congenital heart defects** | | **Patients without congenital malformation** | | **Std_Diff** |
| --- | --- | --- | --- | --- | --- | --- | --- |
| **Number/percentage of patients** | | | 4,880 | 100.00% | 316,910 | 100.00% | . |
| **Age at diagnosis (month)** | | **[0-1]** | 3,841 | 78.71% | . | . | . |
|  |  | **]1-36]** | 1,039 | 21.29% | . | . | . |
| **Death (day of life)** | | **N** | 520 | 10.66% | 4,002 | 1.26% | 0.69 |
|  |  | **≤ 30** | 370 | 71.15% | 3,804 | 95.05% |  |
|  |  | **31-365** | 132 | 25.38% | 114 | 2.85% |  |
|  |  | **> 365** | 18 | 3.46% | 84 | 2.10% |  |
| **Palliative care** | | **Yes** | 190 | 3.89% | 147 | 0.05% | 0.28 |
|  |  | **No** | 4,690 | 96.11% | 316,763 | 99.95% |  |
| **Sex** | | **Male** | 2,485 | 50.92% | 160,392 | 50.61% | 0.01 |
|  |  | **Female** | 2,395 | 49.08% | 156,518 | 49.39% |  |
| **Term of birth (gestation week)** | | **< 24** | 22 | 0.68% | 1,025 | 0.37% | 1.02 |
|  |  | **24-36** | 1,556 | 48.02% | 21,922 | 8.00% |  |
|  |  | **> 36** | 1,662 | 51.30% | 251,061 | 91.63% |  |
| **Birth weight (g)** | | **< 500** | 23 | 0.71% | 819 | 0.30% | 1.03 |
|  |  | **500-1999** | 1,293 | 39.91% | 7,527 | 2.75% |  |
|  |  | **2000-3999** | 1,844 | 56.91% | 256,192 | 93.50% |  |
|  |  | **≥ 4000** | 80 | 2.47% | 9,470 | 3.46% |  |
| **Mother's age at birth (years)** | | **< 18** | 69 | 2.43% | 7,143 | 2.72% | 0.13 |
|  |  | **18-30** | 1,531 | 53.93% | 147,923 | 56.25% |  |
|  |  | **31-39** | 1,000 | 35.22% | 93,299 | 35.48% |  |
|  |  | **≥ 40** | 239 | 8.42% | 14,624 | 5.56% |  |
| **Mother's parity** | | **Primiparity** | 1,583 | 55.76% | 110,927 | 42.18% | 0.27 |
|  |  | **Multiparity** | 1,256 | 44.24% | 152,062 | 57.82% |  |
| **Birth mode** | **Mode of delivery** | **Cesarean** | 1,129 | 39.77% | 46,501 | 17.68% | 0.50 |
|  |  | **Vaginal birth** | 1,710 | 60.23% | 216,488 | 82.32% |  |
|  | **Instrumental birth** | **Yes** | 132 | 4.65% | 17,664 | 6.72% | 0.09 |
|  |  | **No** | 2,707 | 95.35% | 245,325 | 93.28% |  |
| **Medically assisted reproduction** | | **Yes** | 16 | 0.56% | 1,332 | 0.51% | 0.01 |
|  |  | **No** | 2,823 | 99.44% | 261,657 | 99.49% |  |
| **P00.0** Foetus and newborn affected by maternal hypertensive disorders | | | 594 | 12.17% | 12,091 | 3.82% | 0.31 |
| **P01.5** Foetus and newborn affected by multiple pregnancy | | | 416 | 8.52% | 3,443 | 1.09% | 0.35 |
| **P05.0** Light for gestational age | | | 1,014 | 20.78% | 19,505 | 6.15% | 0.44 |
| **P70.0** Syndrome of infant of mother with gestational diabetes | | | 366 | 7.50% | 18,166 | 5.73% | 0.07 |
| **P70.1** Syndrome of infant of a diabetic mother | | | 148 | 3.03% | 2,940 | 0.93% | 0.15 |
| **P96.1** Neonatal withdrawal symptoms from maternal use of drugs of addiction | | | 9 | 0.18% | 63 | 0.02% | 0.05 |
