# Supplementary figures and images for: Epidemiology of congenital heart defects in France from 2013 to 2022 using the PMSI-MCO (French Medical Information System Program in Medicine, Surgery, and Obstetrics) database
Source: PLoS One. 2024 Apr 16;19(4):e0298234. doi: 10.1371/journal.pone.0298234 (PMC11020754; doi:10.1371/journal.pone.0298234)

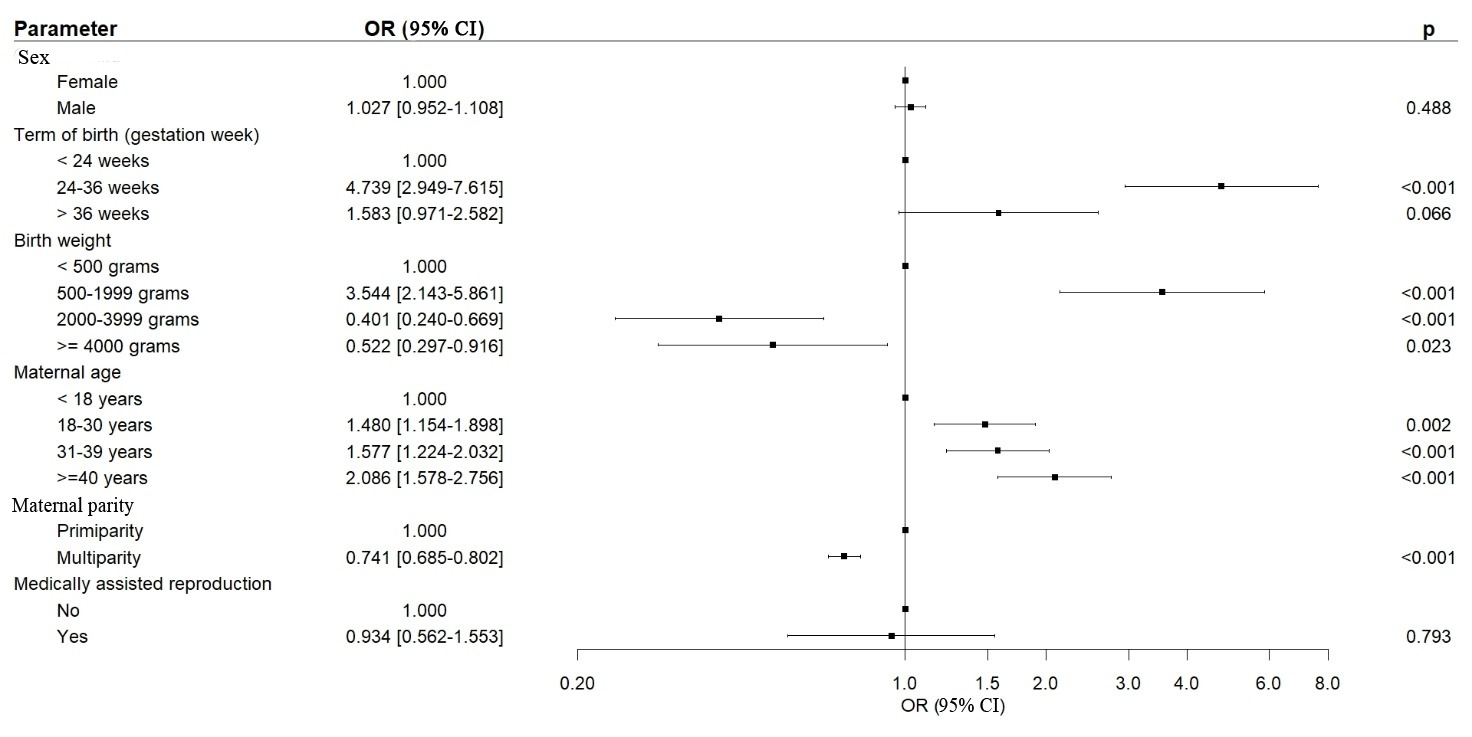

Supplement: S1 Fig — Children with congenital heart defects are compared with the reference population from 2013 to 2022. OR: odds ratio; CI: confidence interval. (TIF) [file pone.0298234.s003.tif]
