# Supplementary material for: Epidemiology of congenital heart defects in France from 2013 to 2022 using the PMSI-MCO (French Medical Information System Program in Medicine, Surgery, and Obstetrics) database
Source: PLoS One. 2024 Apr 16;19(4):e0298234. doi: 10.1371/journal.pone.0298234 (PMC11020754; doi:10.1371/journal.pone.0298234)
Supplement: Attachment — Submitted filename: French CHD epidemiological data - Review note.docx [file pone.0298234.s004.docx]

**Abstract**

The authors have written abstracts very well. However, the PMSI-MCO abbreviation does not match with the French Medical Information System Program in Medicine, Surgery, and Obstetrics.

**Introduction**

The first two paragraphs of the introduction are written appropriately. However, the rest of the introduction paragraphs are written very poorly. Authors need to paraphrase whole paragraphs 3,4 and 5. The authors have described flaws in the database but need to describe the strength of PMSI as well to support why authors have used PMSI.

**Method**

What is reference methodology MR-005? Please describe in detail.

Please explain why this study has an anonymous database and does not involve human subjects. It seems like authors know database and database does have human subject information. Please clarify this.

**Study population and data sources**

Were there confirmed diagnoses of CHD among fetal death and termination of pregnancy cases?

Why did the authors need to collect and analyze data from the French Overseas Departments or regions and Overseas collectivities?

On page 5, line 124: It should frequency of different CHDs. There should not be an association of different CHDs.

On page 6, line 135: What does it mean to prioritize the diagnosis of TGA and ToF in the study?

**Statistical analysis**

The authors presented data on the children’s age at the time of diagnosis. In their study population they had included children who were <= 2 years of age however, in their results section authors presented data of children who were diagnosed with CHD up to 3 years of age.

Per the authors, multiple births were included so mothers were included more than once which makes maternal data analyses completely wrong due to double counting.

Page 7, line 151: for above reference.

Table 2: Represent association of CHD however, it seems like the authors did not show any appropriate statistical analyses to present the association of CHD.

Overall, the Manuscript demonstrates lots of grammatical and improper formation of sentences. The study design and method are not adequately formed to present epidemiological data. There is a huge discrepancy in inclusion criteria and study results which means this study is not designed and presented correctly. This study needs thorough revision in design, study population, and analysis in the future.
